# Elucidating and Mitigating Instabilities of Poly(vinyl alcohol) Thin Films in Aqueous Environments

**DOI:** 10.1021/acs.langmuir.5c04084

**Published:** 2025-10-07

**Authors:** Sophia M. Lee, Jeannie Ji-Ying Tsou, Maya Evans, Carlyn Danese, Yichu Xu, Mahira Mim, Wei Chen

**Affiliations:** Chemistry Department, Carr Laboratory, 7397 Mount Holyoke College, 50 College Street, South Hadley, Massachusetts 01075, United States

## Abstract

In this study, 88% and 99% hydrolyzed poly­(vinyl alcohol) (PVOH^88%H^ and PVOH^99%H^, respectively) polymers were statically adsorbed and spin coated from an aqueous solution onto high molecular weight (HMW) polydimethylsiloxane (PDMS) substrates. The resulting PVOH thin films are unstable and rupture into fractal structures in a diffusion-limited aggregation fashion upon drying. The dynamics of these fractal thin films upon immersion in water and upon exposure to a single water droplet were closely examined. A newly developed “landmarking and overlaying” method was used to quantify the extent of polymer rearrangement under these conditions. Overall, both types of PVOH films exhibit instability in aqueous environments; however, PVOH^88%H^ has faster desorption–readsorption kinetics at the substrate–solution and substrate–solution–air interfaces, resulting in more significant rearrangements upon water exposure. *Ex situ* cross-linking reactions using succinyl chloride in the vapor phase were carried out on the PVOH fractal thin films. Under the optimal reaction conditions, the PVOH fractal structures were entirely preserved upon water exposure. *In situ* cross-linking reactions using glutaraldehyde were performed on the PVOH thin films in contact with solution. Microscopic dewetting of PVOH on HMW PDMS and nanoscopic dewetting of PVOH on intermediate MW PDMS were eradicated. The *in situ* cross-linking results provide convincing evidence that PVOH dewetting takes place during the drying process and can be mitigated.

## Graphical abstract

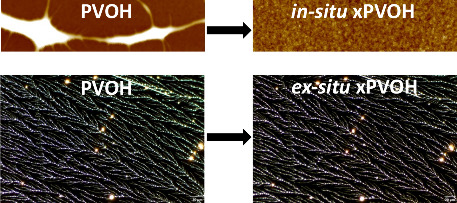

## Introduction

Polymer thin films can impart desirable interfacial properties to underlying substrates, and they are relevant in science, technology, and everyday life. Critically, film morphologies can play an integral role in their functionality. While continuous, pinhole-free thin films are often desirable for applications that necessitate the complete transformation of surface properties,^1^ discontinuous films have become increasingly important in many areas, such as lithography, nanoscience, and biotechnology.^2–6^

The thermodynamic stability of a thin film dictates its morphology and has been extensively studied and reviewed.^1,2,7–17^ Specifically, the effective interface potential, ϕ­(h), is defined as the excess free energy per unit area that is necessary to bring solid–liquid and liquid–gas interfaces from infinity to thickness *h*.^14^ When ϕ­(h) is positive in the entire thickness range, a polymer–substrate system is considered stable.^14^ A system is unstable when there is a global minimum in ϕ­(h).^14^ A metastable system has the combined characteristics of the unstable (at low film thicknesses) and the stable (at large film thicknesses) regimes.^14^ Accordingly, continuous, discontinuous, and both types of thin films can be fabricated in stable, unstable, and metastable systems, respectively. Film dewetting, or rupture, in unstable and metastable systems can be harnessed to produce surface patterns from the nanoscopic to the microscopic scales.^15,17^ However, if continuous films are aspired in these systems, strategies to eradicate film dewetting are requisite. We are particularly interested in hydrophilic polymer thin films fabricated from aqueous solution because aqueous processing is devoid of the use of environmentally harmful organic solvents. However, it is more challenging to derive continuous films of this type due to the destabilizing polar interactions that occur during water evaporation.^12^ Another important consideration is the integrity of hydrophilic polymer thin films in their applications, especially in aqueous environments. Hydrophilic polymers are water-soluble by nature. Developing methodologies to enhance film stability and preserve film morphology in aqueous environments is paramount to improving the functionality of these thin films.

Fractal structures are one type of discontinuous morphology that can be identified in unstable thin films. They are repeated in multiple length scales, very often in a statistical, nonexact fashion. Fractal growth and structures are ubiquitous in nature and can be found in trees, neurons,^18^ river systems,^19^ and Martian araneiforms.^20^ In the early 1980s, Witten and Sander proposed the diffusion-limited aggregation (DLA) model of fractal formation. The model assumes that the kinetic growth of clusters starts from a center point and involves random-walk diffusion and irreversible addition of new particles to the edges of the existing, immobile structures with diffusion being the rate-limiting step.^21,22^ The highly divergent nature of fractal morphology is the result of the exposed ends being more prone to new particle addition than the sites nearer to the center of the cluster. Fractal structures are often characterized by fractal dimension (D)^23,24^ and lacunarity (L)^24,25^ using the box counting method.^26^ Fractal dimension is derived from the number of boxes that a fractal structure occupies in different grid systems; thus, a fractal structure with a high surface coverage has a large D value. Conversely, fractal lacunarity describes the texture or the contribution of gaps in the morphology. A heterogeneous fractal structure with many gaps has a large L value.

We have been interested in fabricating poly­(vinyl alcohol) (PVOH) thin films on polydimethylsiloxane (PDMS) substrates.^27–30^ Both polymers are used in a wide range of applications owing to their unique properties. For example, PDMS has an extremely low glass transition temperature of −123 °C.^31^ It is a liquid at room temperature. Its hydrophobic nature, however, is often undesirable, and manifests in poor wetting, weak adhesion and nonspecific protein adsorption. On the other hand, PVOH is water-soluble and can spontaneously adsorb to hydrophobic substrates from aqueous solution.^32–35^ PVOH polymers are prepared by hydrolysis of poly­(vinyl acetate) and exhibit tunable hydrophobicity and crystallinity, e.g. 88% hydrolyzed PVOH (PVOH^88%H^) is more hydrophobic while 99% hydrolyzed PVOH (PVOH^99%H^) is more crystalline. PVOH thin films fabricated from aqueous solution have been explored to increase the hydrophilicity of PDMS substrates.^27,28,36^ However, the liquid-like character of PDMS destabilizes the PVOH films at the substrate–film interface.^27^ Recently, we demonstrated that the adsorbed PVOH films exhibit a stronger tendency to dewet as the PDMS molecular weight increases or the PDMS layers become thicker.^27,30^ Specifically, PVOH films are metastable on low molecular weight (LMW) and intermediate molecular weight (MMW) PDMS substrates, and form nanoscopically dewetted structures at low film thicknesses.^30^ On high molecular weight (HMW, MW ≥ 9 kDa) PDMS substrates (thickness ≥ ∼ 4 nm), PVOH films are unstable – the ruptured PVOH^88%H^ films consist of droplets while the dewetted PVOH^99%H^ films are composed of fractal branches.^27,30^ The difference in the dewetted morphologies of the two types of PVOH films is attributed to their difference in crystallinity.^27^

In this study, the dynamics of the PVOH^88%H^ and PVOH^99%H^ fractal thin films on HMW PDMS substrates in aqueous environments were closely examined. A semiquantitative image analysis method was developed to determine the extent of polymer rearrangement. *Ex situ* cross-linking reactions using succinyl chloride in the vapor phase were performed to preserve the native PVOH fractal morphologies against rearrangement in water. *In situ* cross-linking reactions using glutaraldehyde in solution were carried out to mitigate PVOH dewetting and rearrangement.

## Experimental Section

### Materials

Silicon wafers (100 orientation, P/B doped, resistivity 1–10 Ω·cm, thickness 475–575 μm) were purchased from International Wafer Service. Trimethylsilyl-terminated (M.W. = 2 kDa, 28 kDa, and 49 kDa) polydimethylsiloxanes (PDMS^2k^, PDMS^28k^, and PDMS^49k^) and silanol-terminated (M.W. = 43.5 kDa) polydimethylsiloxane (SPDMS^44k^) were purchased from Gelest. Poly­(vinyl alcohol) polymers (PVOH^99%H^: M.W. = 89–98 kDa and 99+% hydrolyzed; PVOH^88%H^: M.W. = 85–124 kDa and 88% hydrolyzed), glutaraldehyde solutions (25%), and succinyl chloride were obtained from Sigma-Aldrich. Sulfuric acid (95%) was from Fisher Scientific. HPLC-grade organic solvents were purchased from Pharmco. Water was purified using a Millipore Milli-Q Biocel System (Millipore Corp., resistivity ≥ 18.2 MΩ·cm). Oxygen (99.999%) and nitrogen (99.998%) gases were purchased from Airgas and Ivey Industries, respectively. Glassware was cleaned in a base bath (potassium hydroxide in isopropyl alcohol and water), rinsed with deionized water, and stored in an oven at 110 °C. Prepared samples were stored in a CaSO_4_ desiccator overnight until further use.

### Instrumentation

Silicon wafers were cleaned in a Harrick PDC-001 plasma cleaner. Spin coating was carried out using a Laurell WS-650MZ-23NPPB spin coater. Dynamic light scattering (DLS) measurements were acquired using a Malvern Zetasizer Nano-S equipped with a 4 mW He–Ne laser (λ = 632.8 nm) to determine the size of PVOH chains in solution. Refractive indices of PVOH (n = 1.520) and water (n = 1.330) as well as viscosity of water (η = 0.8872) at 25 °C were assigned. Contact angles were measured using a Ramé-Hart telescopic goniometer with a Gilmont syringe and a 24-gauge flat-tipped needle. Dynamic advancing (θ_A_) and receding (θ_R_) angles were captured by a camera and digitally analyzed while Milli-Q water in the syringe was added to and withdrawn from the drop, respectively. The standard deviations of the reported contact angle values are less than or equal to 2° unless otherwise specified. Native silicon dioxide and polymer layer thicknesses were measured using a Gaertner Scientific LSE Stokes ellipsometer at a 70° incident angle (from the normal to the plane). The light source is a He–Ne laser (λ = 632.8 nm). Thicknesses were calculated using the following refractive indices: air, n_o_ = 1; silicon oxide and polymer layers, n_1_ = 1.46; silicon substrate, n_s_ = 3.85 and *k*_s_ = −0.02 (absorption coefficient). Measurement error is within 1 Å as specified by the manufacturer. Each reported thickness and contact angle value is an average of at least eight measurements obtained from at least four samples from two different batches and two readings from different locations on each sample. Nanoscopic surface topography was imaged using a Veeco Metrology Dimension 3100 atomic force microscope (AFM) with a silicon tip operating in tapping mode. Roughness and section analyses of surface features were determined using Nanoscope software. Microscopic morphology of PVOH thin films was characterized using an Olympus BX51 optical microscope in darkfield reflective mode. Variations in hues exhibited in optical images are artifacts caused by the instrument and software settings. Multiple AFM and optical images from different samples of the same type and different locations on each sample were captured; representative images were chosen.

### Static Adsorption and Spin Coating of PVOH Thin Films on PDMS Substrates

The procedures for chemically attaching PDMS polymers to silicon wafers and physically adsorbing PVOH polymers to PDMS substrates were adapted from the published work.^30,37^ In brief, silicon wafers (1.4 cm × 1.4 cm) were cleaned by oxygen plasma at ∼ 300 mTorr and 30 W for 15 min. PDMS polymers were covalently attached to the wafers via reactions at 100 °C for 24 h. 400 μL of 0.1 wt % PVOH solution was dispensed onto a PDMS substrate and left to adsorb for 1 min. In a static adsorption trial, the sample was rinsed with 1 mL of Milli-Q water using a micropipette, and immediately dried under a stream of nitrogen gas. In an adsorptive spin coating trial, the sample was spun at a desired rate (900 to 6000 rpm) for 1 min under nitrogen.

### *In Situ* Cross-Linking of PVOH Using Glutaraldehyde

Glutaraldehyde (25%) and sulfuric acid were quickly mixed in a 20:1 volume ratio in a 1.5 mL centrifuge tube. The solution was used within 15 min of preparation. 300 μL of PVOH solution was dispensed onto a PDMS substrate. After a 1 min adsorption period, 105 μL of the cross-linking solution was carefully added to the PVOH drop without overflowing. The cross-linking reaction was then carried out for a desired period of time (1, 5, or 10 min) before the sample was thoroughly rinsed with Milli-Q water and dried under a nitrogen stream.

### *Ex Situ* Cross-Linking of PVOH Using Succinyl Chloride

A PVOH-containing sample was placed in a scintillation vial. ∼ 20 μL of succinyl chloride was carefully added without direct contact with the sample. Each sample was heated at a desired temperature for a desired amount of time.

### Solvent Annealing

PVOH samples, before or after cross-linking, were submerged in Milli-Q water at room temperature. After 1 h, the samples were dried under a nitrogen stream.

### Interfacial Dynamics of PVOH Thin Films upon Water Exposure

Thirty μL of Milli-Q water was deposited onto the center of a PVOH sample, before or after cross-linking. An image of the drop was captured every 5 min using the contact angle goniometer until water completely evaporated after ∼ 2 h. Drop contact angle, width, and height were monitored as a function of evaporation time. A time-lapse video of the evaporating drop was created using iMovie software.

### Optical Image Processing and Analysis

To compare PVOH fractal morphology before and after solvent annealing, Fiji software was used to color code the corresponding optical images before (in magenta) and after (in green) treatment. Photoshop software was used to overlay the color-coded images. The opacity of the after image was decreased to 50%, then sample landmarks (features remained unchanged by the treatment) were matched by placing the after image over the before image at an optimal position and angle. The color-coded images as well as the overlaid images were then binarized in ImageJ. The PVOH^88%H^ samples were binarized using the auto threshold function, while the PVOH^99%H^ samples were binarized manually by selecting a threshold that resulted in continuous fractal outlines. The PVOH^99%H^ images, which have hollow fractals, were filled in using Photoshop software. Images were then cropped using standard coordinates (200, 200, 1520, 800) to remove scale bars and unmatched edges. From binarized images, percent of surface coverage (%C) as well as fractal dimension (D) and lacunarity (L) of PVOH fractal morphologies were determined using the Fraclac plugin in Fiji. Each reported%C, D and L value is an average of at least four measurements from two different samples. In the interfacial dynamics studies, global images across the droplet footprint were obtained by merging individual optical images captured at 50× magnification using Fiji software.

## Results and Discussion

### PVOH Fractal Morphology

Over the course of this work, three types of HMW PDMS substrates, respectively prepared by reacting PDMS^28k^, PDMS^49k^, and silanol-terminated SPDMS^44k^ with silicon wafers, were used due to supply issues. Since they are well within the established HMW regime (MW ≥ 9 kDa) and no detectable differences in their wettability characteristics and adsorbed PVOH thin film behaviors were observed, they are referred to generally as HMW PDMS substrates and considered equivalent. The fractal morphologies of PVOH^88%H^ and PVOH^99%H^ thin films prepared by both static adsorption and spin coating on HMW PDMS substrates are shown in Figure 1. The PVOH thin films were visibly dry within a few seconds using both preparation methods. Spin-coating results in global alignment of the fractal branches in the exit direction.^29^ On the local scales, however, the two preparation methods produce thin films of indistinguishable morphologies and similar ellipsometric thicknesses of ∼ 2 nm. In this work, PVOH thin films were prepared by spin coating unless otherwise specified.

**Figure 1:**
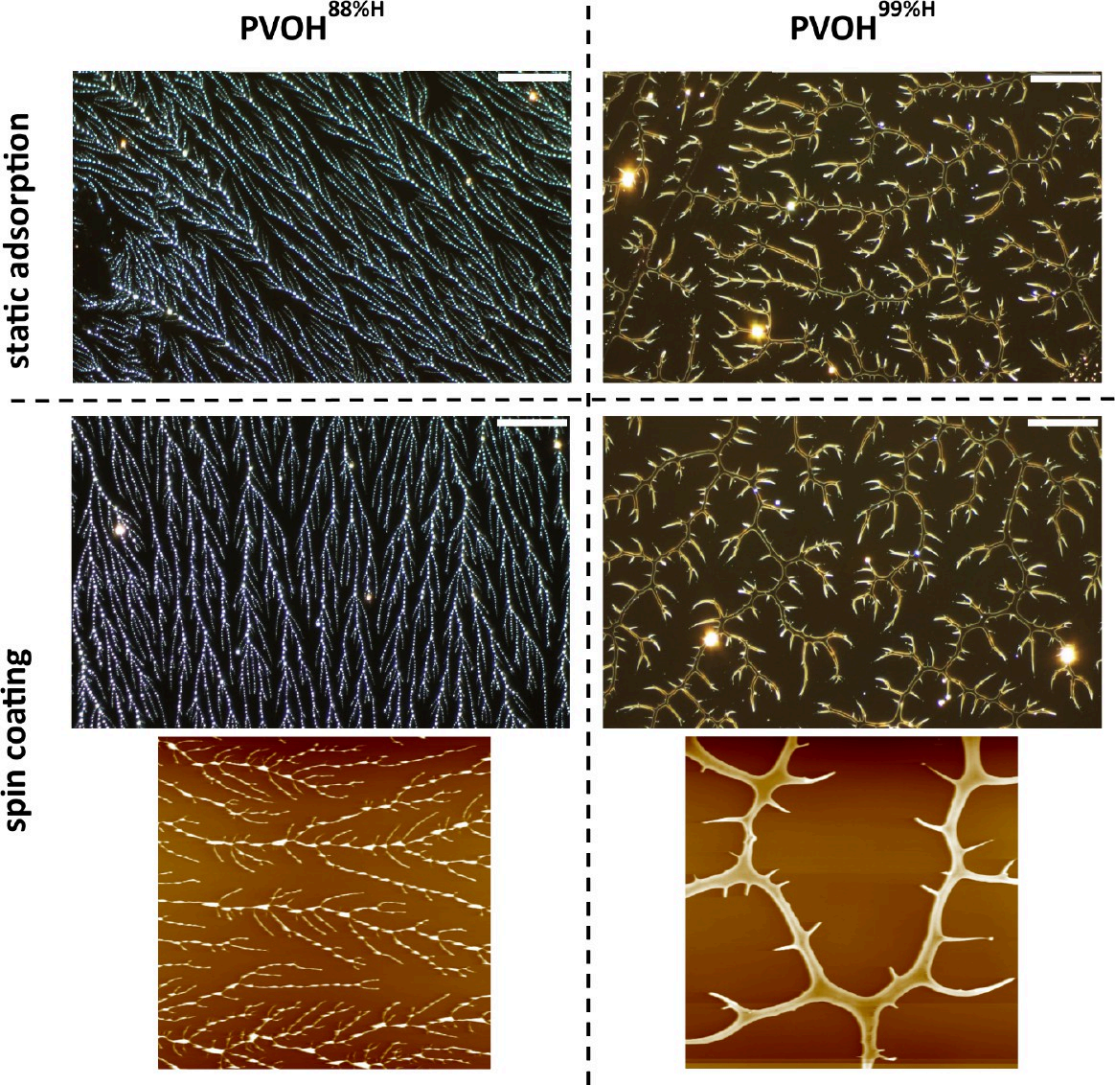
Optical images (top two rows) and AFM images (bottom row: size, 50 μm × 50 μm; data scale, 100 nm) of PVOH^88%H^ (left column) and PVOH^99%H^ (right column) fractal thin films on HMW PDMS. Top row images are PVOH samples prepared by static adsorption. Middle and bottom row images are PVOH samples prepared by spin coating at 6000 rpm. Scale bars represent 50 μm.

Figure 1 reveals prominent distinctions between PVOH^88%H^ and PVOH^99%H^ fractal features. While the PVOH^88%H^ fractals consist of a multitude of droplets that form thin, straight, and densely packed branches, the PVOH^99%H^ fractals are comprised of continuous, wide, curvy, and loosely packed branches. Their key characteristics are quantified and compared in Table 1. Intriguingly, the main fractal branches of PVOH^88%H^ and PVOH^99%H^ are comparable in height even though the former are significantly narrower. In conjunction with the fact that both types have similar ellipsometric thicknesses and surface coverages, the formation mechanism of the fractal features is consistent with the diffusion-limited aggregation model in two dimensions. Since *in situ* optical imaging did not reveal any detectable features at the substrate–solution interface, dewetting presumably takes place during the drying process.^27^ Illustrations of the PVOH adsorption and aggregation are shown in Figure 2 (a and b). During the evaporation of water, the PVOH chains at the substrate–solution interface are forced into proximity. Intermolecular attractions—mostly hydrogen bonding for PVOH^99%H^ and hydrophobic interactions for PVOH^88%H^ ^27,30^ —drive polymer aggregation. The presence of droplets along the fractal backbone in the PVOH^88%H^ films (Figure 1 left column) is consistent with PVOH^88%H^’s lower ability to hydrogen bond and the droplet morphology observed in amorphous polymer films due to Rayleigh instabilities.^8^ Since polymer diffusion is facilitated by the presence of water, the wider branches of PVOH^99%H^ are attributed to the polymer’s capability to retain water and stay mobile for a longer time. The correlation between the wider branches and the enhanced polymer chain mobility is confirmed by the simulation study.^38^ Overall the PVOH^88%H^ fractal morphology has a larger fractal dimension (D) and a lower fractal lacunarity (L) (Table 1), consistent with its slightly higher surface coverage and more uniform distribution, respectively. Lastly, in terms of wettability, advancing and receding water contact angles on the HMW PDMS substrates are 109 ± 2° and 95 ± 2°, respectively. The low coverage of the hydrophilic PVOH branches on the hydrophobic PDMS substrates results in minimal change in the advancing water contact angles. However, the hydrophilic PVOH branches pin receding water droplets and significantly lower the receding contact angles. The large standard deviations reflect the heterogeneous nature of the composite surfaces.

**Table 1:** Comparison of PVOH^88%H^ and PVOH^99%H^ Fractal Thin Films Spin Coated on HMW PDMS: Main Fractal Widths, Main Fractal Heights, Ellipsometric Thicknesses, Surface Coverages (%C), Fractal Dimensions (D), Fractal Lacunarities (L), and Advancing and Receding Water Contact Angles (θ_A_/θ_R_)

	width (μm)	height (nm)	thickness (nm)	%C	D	L	θ_A_/θ_R_ (deg)
88%H	0.3 ± 0.1	53 ± 16	1.8 ± 0.7	6.8 ± 1.0	1.75 ± 0.02	0.26 ± 0.03	112 ± 3/64 ± 8
99%H	6 ± 3	36 ± 4	2.2 ± 0.9	5.8 ± 1.3	1.64 ± 0.03	0.44 ± 0.05	115 ± 2/69 ± 11

**Figure 2:**
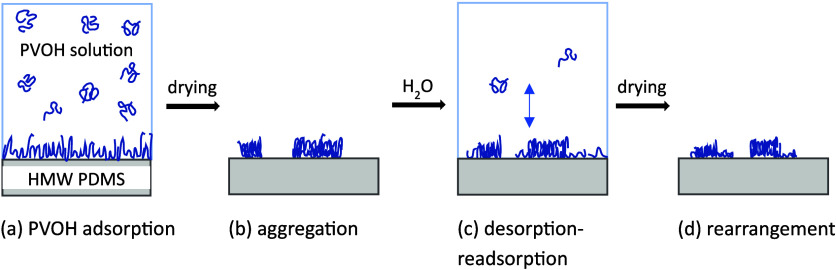
Illustrations of PVOH dynamics on HMW PDMS. (a) PVOH spontaneously adsorbs to the HMW PDMS substrate from aqueous solution. (b) Drying causes PVOH dewetting and aggregation. (c) Upon re-exposure to water, PVOH undergoes desorption–readsorption equilibrium. Desorption takes place from aggregated features, and readsorption occurs on the exposed substrate. (d) Drying causes secondary PVOH dewetting and aggregation resulting in PVOH rearrangement relative to panel b.

### PVOH Dynamics upon Water Exposure

Stability, especially in aqueous environments, is of paramount consideration in materials applications. In order to investigate this critical material property, the PVOH^88%H^ and PVOH^99%H^ fractal thin films supported on HMW PDMS substrates were immersed in water at room temperature for 1 h followed by rapid drying (a few seconds) using a stream of nitrogen gas. Optical images taken before and after this treatment are shown in the first two rows of Figure 3. While the PVOH^88%H^ film completely loses its resemblance to the original, the PVOH^99%H^ film retains most of its native features. After water immersion and drying, the notable changes in PVOH^99%H^ include the thinning of the main branches and the appearance of new, fine branches. The appearance of the fine branches in the PVOH^99%H^ films is attributed to the desorption-readsorption dynamics and the secondary dewetting of a small amount of readsorbed PVOH^99%H^ on the PDMS substrates upon drying. These processes are illustrated in Figure 2 (c and d). It is worth noting that similar results were attained when the PVOH films were only exposed to water for 1 min (not shown). This indicates that fast PVOH desorption-readsorption dynamics and rapid drying lead to new PVOH morphologies that are kinetically trapped states.

**Figure 3:**
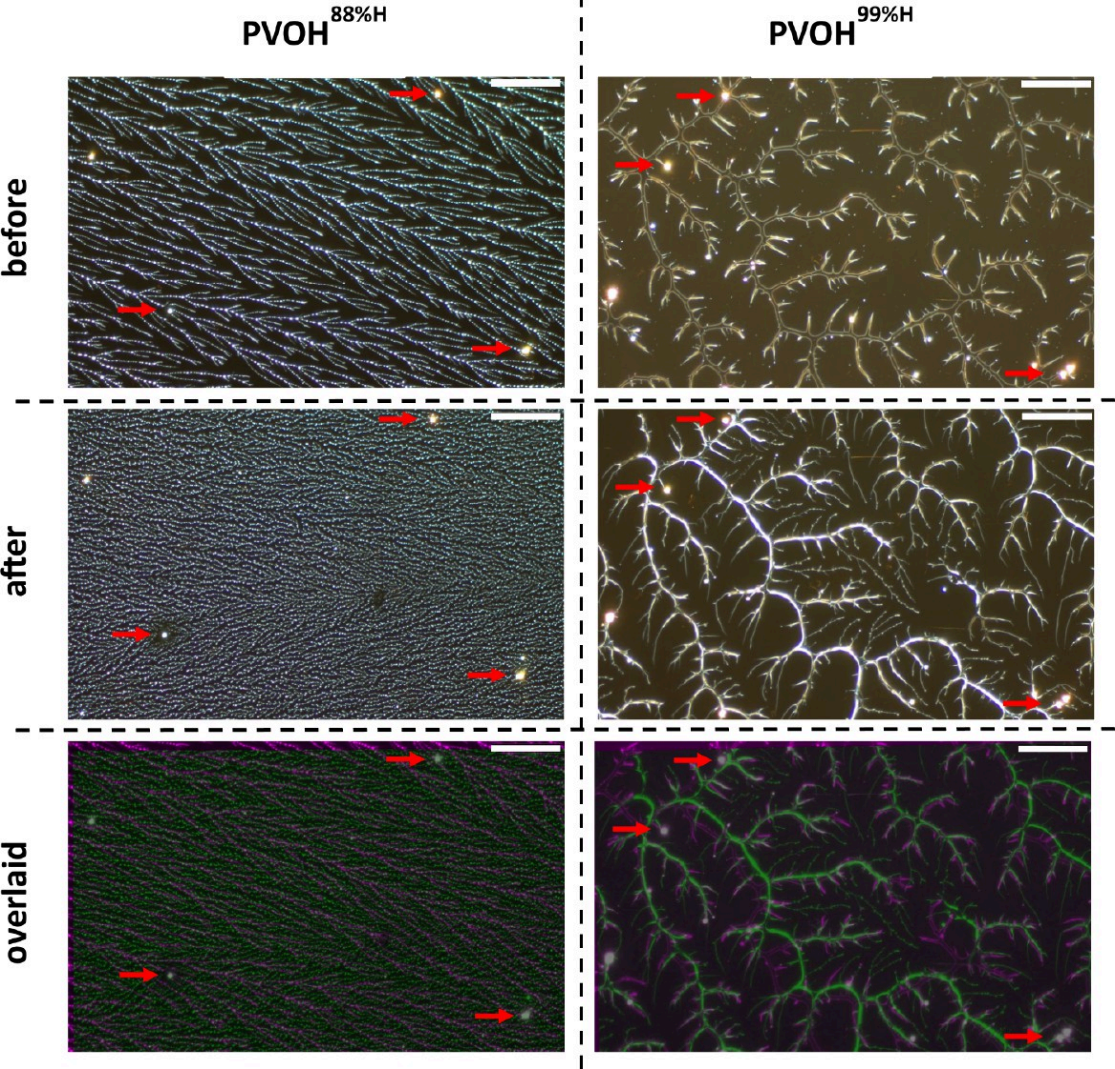
Optical images of spin-coated PVOH^88%H^ (left column) and PVOH^99%H^ (right column) fractal thin films on HMW PDMS before and after water immersion at room temperature for 1 h. First row images show PVOH features before water immersion. Middle row images show PVOH features after water immersion. Bottom row images are overlaid images before (colored magenta) and after (colored green) water immersion. Landmarks, as indicated by red arrows, were used to identify the same sample areas before and after water immersion and to facilitate image overlaying process. Scale bars represent 50 μm.

Interestingly, water immersion resulted in insignificant changes in terms of PVOH thickness (Figure 1S), surface coverage (Figure 2S), and fractal dimension and lacunarity (Figure 3S) within the standard deviations. This indicates that there is a negligible net loss of PVOH during the process of desorption and readsorption. This dynamic polymer rearrangement on surfaces is facilitated by water and is an example of two-dimensional “solvent annealing.”

In addition to visual assessment, we developed a semiquantitative method – “Landmarking and Overlaying” – for image analyses. Landmarks or distinct surface features were used to locate the same sample areas for imaging before and after water immersion and to facilitate the image overlaying process. The after image was placed on top of the before image and set to 50% transparency so that both images are equally weighed in the final overlaid image, as shown in the bottom row of Figure 3. To quantify the extent of polymer rearrangement, %R is defined in eq 1. For PVOH^88%H^ and PVOH^99%H^ fractal thin films, the extents of rearrangement were determined to be 51 ± 13% and 29 ± 9%, respectively. The greater propensity of the PVOH^88%H^ fractal structures to rearrange is consistent with the trend based on the simulation results from an earlier work.^39^ The higher crystallinity of the PVOH^99%H^ thin films contributes to their greater aqueous stability.

$$\%R=\frac{\%C\,\;\mathrm{overlaid image}-\mathrm{\%C before image}}{\mathrm{\%C after image}}$$ 1

To examine time-dependent PVOH desorption-readsorption dynamics, 30 μL water was deposited on PVOH fractal thin films. Drying took a much longer time, ∼ 2 h, compared to the solvent annealing experiments. A 30 μL drop (∼5 mm in diameter on a 14 mm × 14 mm sample) allows for explicit visualization of the three distinct regions (unperturbed, perturbed, and interface) as shown in Figure 4. The global image and the higher resolution images of the drop edge of a PVOH^88%H^ sample (Figure 4 top) depict a bright unperturbed background, a distinct interface, and a mostly dark interior with a bright center spot in the drop region. The thickness profile (Figure 4S) across the drop footprint indicates a thickness decrease of 2–3 nm at the edge and a spike with a thickness increase of ∼ 10 nm in the center. This indicates that the dark interior is caused by the complete removal of PVOH^88%H^ polymer, and the bright spot in the center consists of the PVOH^88%H^ mass that was accumulated by the receding water drop.

**Figure 4:**
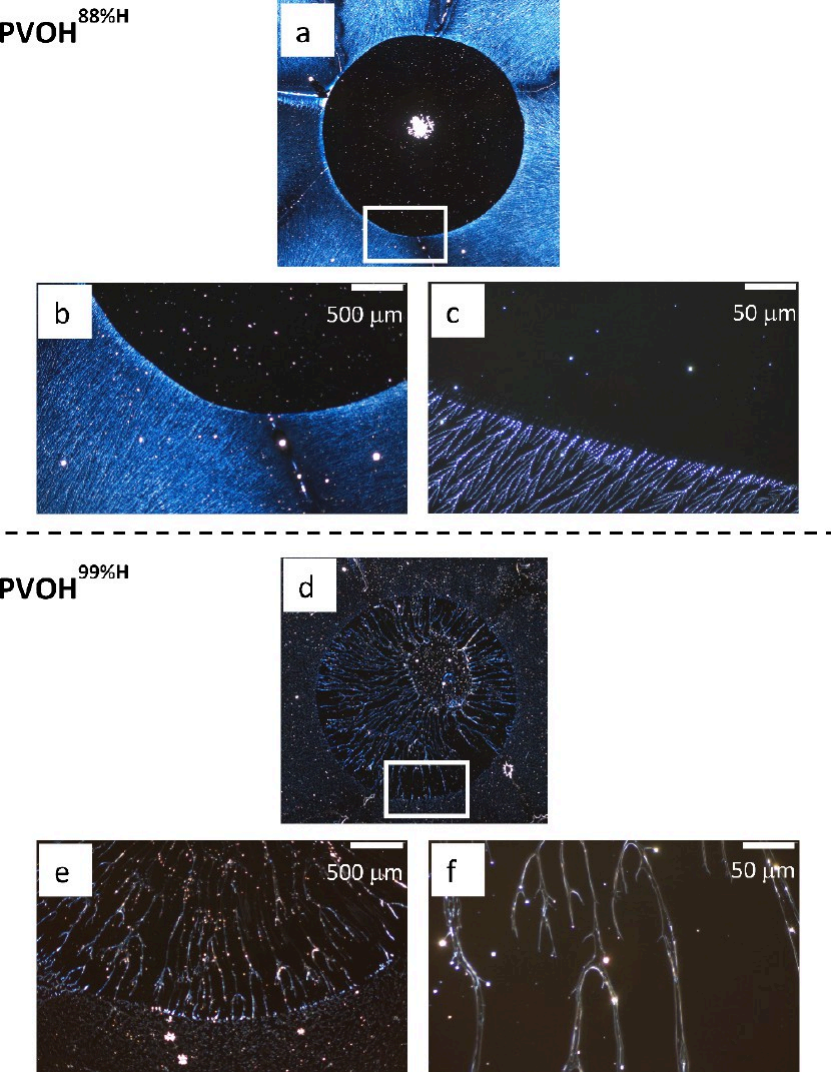
Optical images of PVOH^88%H^ (top) and PVOH^99%H^ (bottom) fractal structures on HMW PDMS after being exposed to a 30 μL water drop. (a and d) Global images, including dried drop regions (5 mm in diameter) surrounded by unperturbed PVOH fractal features. (b, c, e, and f) Higher resolution images captured at drop boundaries (boxed regions in the global images).

The corresponding optical images of a PVOH^99%H^ sample (Figure 4 bottom) tell a different story. Instead of having a PVOH-depleted region in the drop interior, significantly larger fractal features form from the interface extending to the center where some polymer mass accumulation is visible. The thickness profile (Figure 4S) depicts a very slight decrease in thickness at the edge and a thickness spike in the center. Similarly to PVOH^88%H^, the PVOH^99%H^ desorption-readsorption dynamics at the substrate–solution interface and the contracting drop result in detectable mass redistribution from the edge to the center. The key difference is that a significant amount of PVOH^99%H^ remains on the substrates and the desorbed PVOH^99%H^ readsorbs (and crystallizes) onto the existing branches in a process similar to Oswald ripening. The absence of the new, fine branches that appeared in the PVOH^99%H^ structures after solvent annealing (Figure 3) is most likely due to contact line pinning by the existing branches, the significantly longer drying time, and the enhanced stability of the larger PVOH^99%H^ branches. Thermodynamics favors decreasing the surface area of the higher surface energy PVOH structures and increasing the exposure of the lower surface energy PDMS substrates. The accumulation of the PVOH^88%H^ mass in the drop center is also consistent with the thermodynamic considerations.

Time-lapse videos of an evaporating 30 μL water droplet on PVOH^88%H^ and PVOH^99%H^ fractal thin films are in the . Contact angle and drop width profiles as a function of the extent of drying are shown in Figure 5S. At the early stage of evaporation, the drop on PVOH^88%H^ experiences horizontal contraction – constant contact angle with decreasing drop width. This implies that the PVOH^88%H^ desorption-adsorption dynamics are faster than the receding rate of the contact line such that PVOH^88%H^ is removed from the substrate and brought to the center by the receding drop. On the other hand, the drop on PVOH^99%H^ exhibits vertical contraction – decreasing contact angle with constant drop width. The PVOH^99%H^ desorption-adsorption dynamics must be slower than the receding rate of the contact line such that the adsorbed PVOH^99%H^ pins the evaporating drop. The more pliable characteristics of PVOH^88%H^ portrayed in this set of experiments are consistent with its behaviors during solvent annealing, both of which are attributed to the weaker cohesion (lower degree of crystallinity) of the polymer.

### PVOH Stabilization via Cross-Linking

The dynamic behaviors of the PVOH fractal thin films upon water exposure impose concerns in their applications in aqueous media. To gain access to continuous, pinhole-free PVOH structures and to preserve the PVOH fractal structures on HMW PDMS, two cross-linking reactions were carried out as illustrated in Figure 5. An *in situ* cross-linking reaction using glutaraldehyde (GA)^34,40^ was carried out on the adsorbed PVOH layer in solution prior to drying while an *ex situ* cross-linking reaction using succinyl chloride (SC)^41–43^ was performed on the dried PVOH fractal structures in the vapor phase.

**Figure 5:**
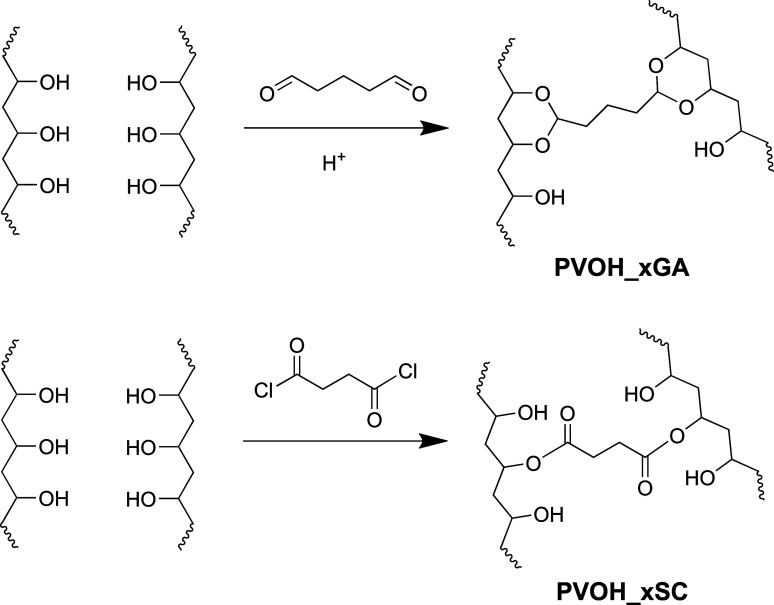
PVOH cross-linking chemistry using glutaraldehyde (GA, top) and succinyl chloride (SC, bottom).

The *in situ* cross-linking reaction using GA was performed immediately after static adsorption such that the adsorbed PVOH layer on HMW PDMS was cross-linked at the solid-solution interface without exposure to air. The optical images of the PVOH_xGA thin films do not have any detectable dewetted features (not shown), which is in stark contrast to the extremely dewetted PVOH control samples (Figure 1). The corresponding AFM images of the PVOH^88%H^_xGA cross-linked for 1, 5, and 10 min are in Table 2. The PVOH^88%H^_xGA thin films appear to be mostly smooth with only a few discernible cracks on the sample that was cross-linked for the shortest amount of time. The PVOH^99%H^_xGA thin films showed similar nanoscopic morphologies (Table 1S).

**Table 2:**
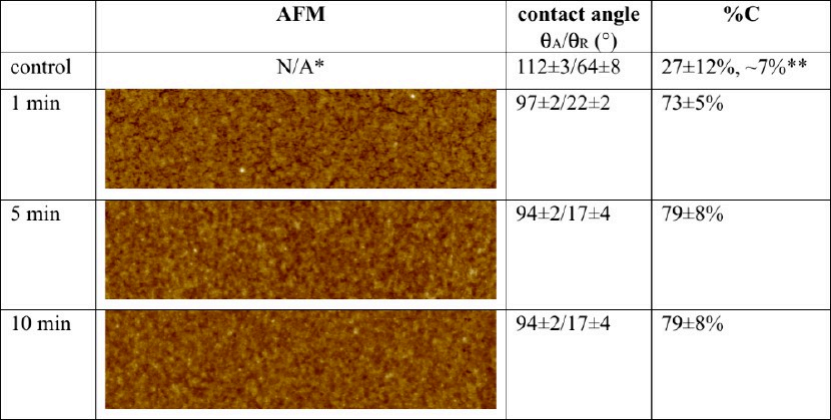
AFM Images (size, 1.25 μm × 5 μm; data scale, 10 nm), Advancing (θ_A_) and Receding (θ_R_) Contact Angles, and Surface Coverages (%C) of Statically Adsorbed PVOH^88%H^ on HMW PDMS after *In Situ* Cross-Linking Using Glutaraldehyde for Various Amounts of Time

* See Figure 1.

** The first value is based on the average contact angle, and the second value is based on image analysis.

To quantify the extent of nanoscopic dewetting, the PVOH surface coverage of the control and the cross-linked samples was calculated using the Israelachvili eq 2.^44^

2 $${\left(1+\cos\;\theta \right)}^{2}=f_{1}{\left(1+\cos\;\theta_{1}\right)}^{2}+f_{2}{\left(1+\cos\;\theta_{2}\right)}^{2}$$

The surfaces were treated as binary composites consisting of f_1_ surface fraction of component 1 (PVOH) and f_2_ surface fraction of component 2 (PDMS) with f_1_ + f_2_ = 1. The PVOH surface coverage was calculated based on the contact angle values of the composite surface (θ), pure component 1 (θ_1_), and pure component 2 (θ_2_). The advancing and receding contact angles (θ_A_/θ_R_) of pure PVOH films and HMW PDMS substrates are 63 ± 2°/17 ± 2°^34^ and 109 ± 2°/95 ± 2°, respectively. Those of the composite surfaces are shown in the second to the last column of Table 2. The average values of the advancing and receding contact angles were used to determine the PVOH surface coverage (f_1_).^30^ On the PVOH control samples, ∼ 27% coverage was calculated based on the average contact angles and the Israelachvili equation. The heterogeneous nature of the PVOH fractal structures contributes to significant uncertainties in the receding contact angle measurements and the surface coverage values. On the other hand, the image analysis method produces a more direct and reliable value of 7% coverage of the PVOH control samples (Figure 1). The PVOH samples become much more homogeneous after cross-linking (Table 2), and thus the surface coverage values obtained using the Israelachvili equation are reasonably dependable. After 1 min cross-linking time, ∼ 73% of the PDMS substrate was covered by the PVOH^88%H^ thin film. After 5 min, the PVOH^88%H^ coverage plateaued at ∼ 79%. It is worth noting that there is undetectable morphology change in the 5 min and longer PVOH_xGA films upon solvent annealing. The *in situ* cross-linked PVOH^99%H^ thin films on HMW PDMS show similar results (Table 1S). The optical images, AFM images, and contact angle values of the control and cross-linked PVOH thin films collectively demonstrate that the *in situ* cross-linking reaction using GA is effective at preventing microscopic dewetting and minimizing nanoscopic dewetting of the adsorbed PVOH thin films on HMW PDMS substrates. This also provides solid evidence that PVOH dewetting and aggregation takes place during drying.

In our earlier work,^30^ it was demonstrated that PVOH thin films are metastable on intermediate molecular weight (MMW) PDMS substrates, such as PDMS^2k^. Specifically, nanoscopic dewetting was observed on statically adsorbed PVOH thin films in this system. Therefore, *in situ* cross-linking using GA was performed on these thin films to evaluate the method’s ability to eliminate nanoscopic dewetting. The AFM images of the control and PVOH^88%H^_xGA on PDMS^2k^ are shown in Table 3. The nanoscopic holes on the control samples completely disappeared on all cross-linked samples. Based on the dynamic contact angles of PDMS^2k^ (θ_A_/θ_R_ = 107 ± 2°/91 ± 2°) and the method illustrated earlier, the PVOH^88%H^ surface coverage increased from 84% to 100%. The *in situ* cross-linked PVOH^99%H^ thin films show a similar trend (Table 2S), i.e. pinhole free PVOH_xGA thin films were attained after 5 min reaction time.

**Table 3:**
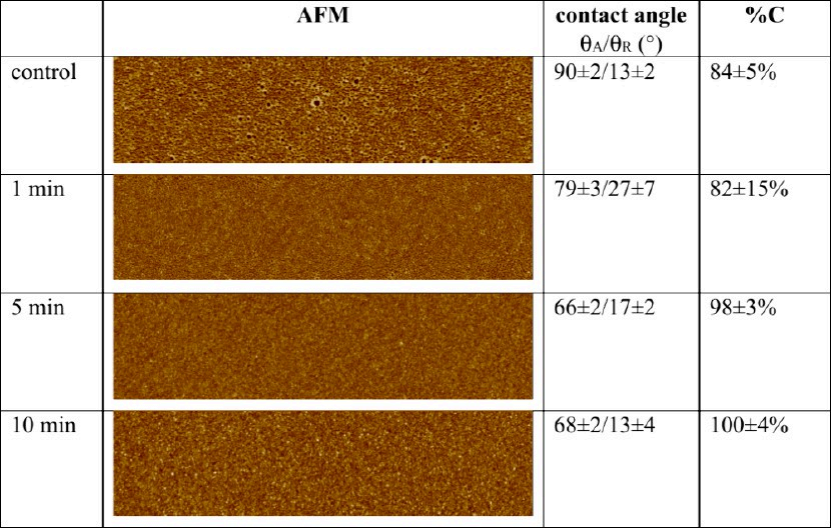
AFM Images (size, 1.25 μm × 5 μm; data scale, 10 nm), Advancing (θ_A_) and Receding (θ_R_) Contact Angles, and Surface Coverages (%C) of Statically Adsorbed PVOH^88%H^ on PDMS^2k^ after *In Situ* Cross-Linking Using Glutaraldehyde for Various Amounts of Time

The *in situ* cross-linking reaction using GA provides convincing evidence that the adsorbed PVOH thin films at the substrate–solution interface are pinhole free and that drying triggers PVOH dewetting and aggregation. While the *in situ* cross-linking reaction is 100% effective at preventing microscopic dewetting, the unstable PVOH films on HMW PDMS have such strong tendency to dewet that this method cannot completely eliminate nanoscopic dewetting. However, dewetting is entirely eradicated in the case of metastable PVOH films on MMW PDMS.

In an attempt to preserve the PVOH fractal structures on HMW PDMS (Figure 1), *ex situ* cross-linking reactions using GA were performed. However, due to the aforementioned fast PVOH desorption-readsorption kinetics in aqueous solution, the PVOH film structures were significantly altered after the reaction. To avoid the competitive PVOH desorption-readsorption dynamics in the presence of water, vapor phase *ex situ* cross-linking reactions using succinyl chloride (SC) were carried out on the PVOH fractal thin films.

To the best of our knowledge, SC has not been reported as a cross-linker for PVOH polymers. The diacid chloride functional groups are anticipated to react readily with two alcohol groups from one or two PVOH polymer chains. In the latter case, PVOH chains are cross-linked together (Figure 5) with reduced mobility and solubility. A significant amount of effort was made to identify the lowest reaction temperature and the shortest reaction time necessary to impart stability to PVOH fractal thin films in aqueous media. In general, the PVOH^88%H^ polymer is more mobile and contains fewer −OH groups, and thus requires more extreme reaction conditions. After the vapor phase reaction with SC at 70 °C for 1 h, the PVOH^99%H^_xSC morphology exhibited undetectable change upon water immersion at room temperature for 1 h (Figure 6S). Considering that the glass transition temperature (*T*_g_) of PVOH is 85 °C^31^ and that the PVOH^99%H^ thin film structures contain water, it is probable that 70 °C is above the effective *T*_g_ of PVOH^99%H^. On the other hand, the PVOH^88%H^_xSC fractal thin films underwent notable rearrangements (Figure 6S and Figure 6a) albeit to a much lesser extent than their un-cross-linked counterparts (Figure 3). When the reaction temperature for PVOH^88%H^ was subsequently raised to 100 °C, which is above the *T*_g_, and the reaction was carried out for 1 h, most of the PVOH^88%H^_xSC features were retained after solvent annealing (Figure 6b). Further increasing the reaction time to 6 h at 100 °C successfully inhibited PVOH^88%H^_xSC rearrangement during solvent annealing (Figure 6c).

**Figure 6:**
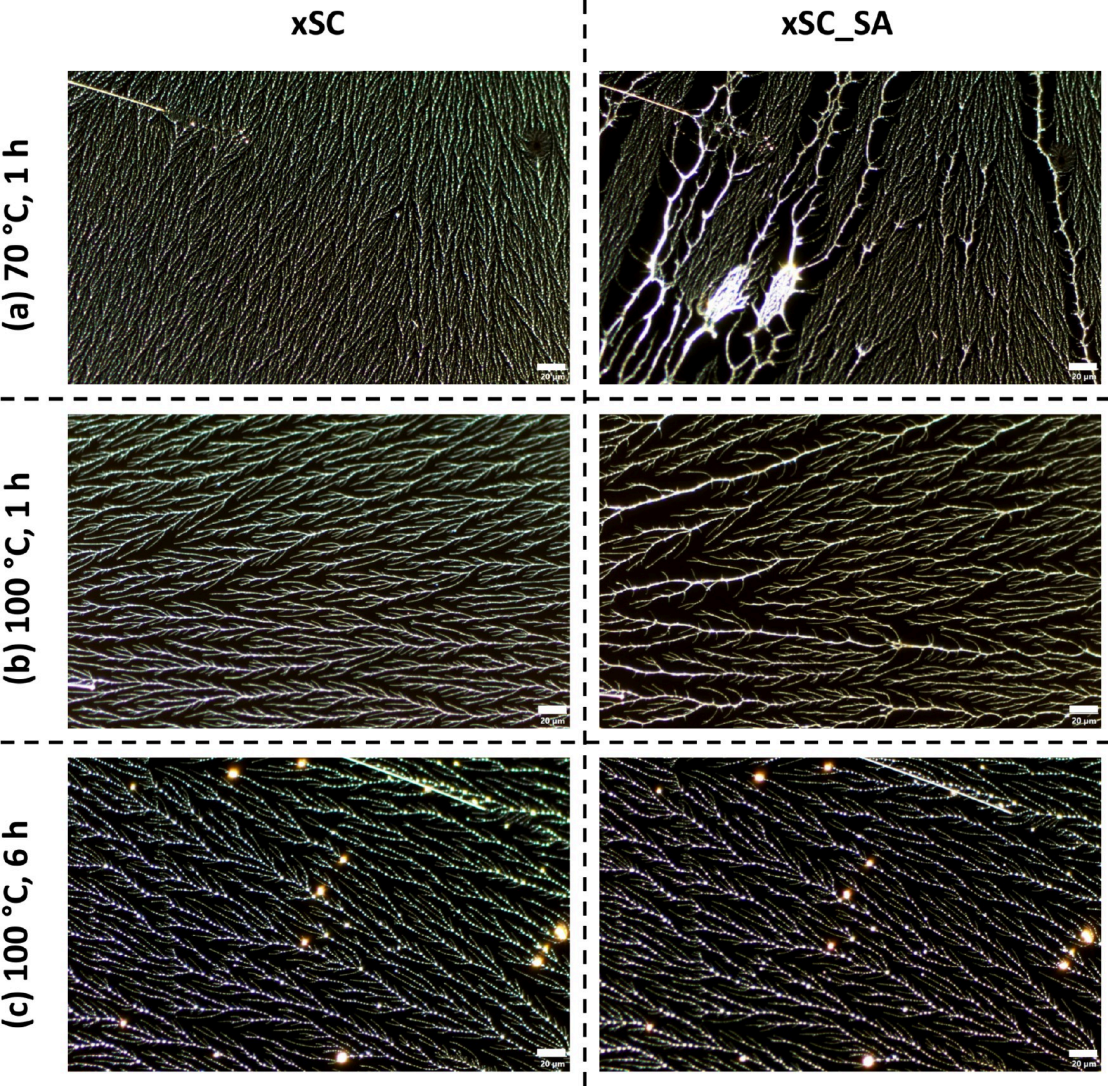
Optical images of spin-coated PVOH^88%H^ on HMW PDMS cross-linking with succinyl chloride before (xSC, left column) and after (xSC_SA, right column) solvent annealing at room temperature for 1 h under different conditions: (a) 70 °C for 1 h (first row), (b) 100 °C for 1 h (second row), and (c) 100 °C for 6 h (third row). Scale bars represent 20 μm.

Succinyl chloride is a novel and effective cross-linker at immobilizing PVOH fractal thin films in aqueous environments. Reactions in the vapor phase offers enormous advantages compared to those in solution. Specifically, the competitive PVOH rearrangement in solution can be entirely avoided and the PVOH fractal structures are seamlessly preserved. It is important to note that vapor phase reactions with solid polymers, such as PVOH thin films, should be performed above the effective glass transition temperature to both afford polymer mobility and allow for the effective diffusion of reagent into the thin film structures.

## Conclusions

In this work, we demonstrated the thermodynamic instability of PVOH thin films on HMW PDMS substrates. Rather than completely wetting the substrate surface, the PVOH polymers aggregate and form fractal structures in a diffusion-limited aggregation fashion during drying, with the final film morphology determined by the rate of water removal. The hydrophilic nature of the resulting PVOH fractal films renders them unstable in aqueous environments. In order to analyze the extent of PVOH rearrangement upon exposure to water, a novel, semiquantitative method was developed. Notably, the PVOH^99%H^ fractal thin films were determined to be more stable in aqueous environments than their PVOH^88%H^ counterparts due to their more crystalline nature. Finally, two cross-linking methods were found to be effective at fabricating continuous as well as discontinuous PVOH thin films that are stable in aqueous environments. The *in situ* cross-linking reaction using the well-established glutaraldehyde chemistry successfully mitigates PVOH dewetting and produces continuous thin films. On the other hand, the *ex situ* reaction using a new cross-linking reagent for PVOH, succinyl chloride, successfully preserves discontinuous fractal structures. These stabilization strategies can potentially be extended to other hydrophilic thin films.

## Supplementary Material







## Notes

Additional optical images, AFM images, and various data (surface coverages, thicknesses, fractal dimensions and lacunarities, and contact angles) of PVOH thin films on different PDMS substrates (PDF)
Video of a water droplet evaporating from a PVOH^88%H^ thin film (MOV)
Video of a water droplet evaporating from a PVOH^99%H^ thin film (MOV)
