# Supplementary material for: Elucidating and Mitigating Instabilities of Poly(vinyl alcohol) Thin Films in Aqueous Environments
Source: Langmuir. 2025 Oct 7;41(41):28119–28. doi: 10.1021/acs.langmuir.5c04084 (PMC12548347; doi:10.1021/acs.langmuir.5c04084)
Supplement: Supplementary file 1 [file la5c04084_si_001.pdf]

Elucidating and mitigating instabilities of poly(vinyl alcohol) thin films in aqueous environments

Sophia M. Lee[$], Jeannie Ji-Ying Tsou[$], Maya Evans, Carlyn Danese, Yichu Xu, Mahira Mim, and Wei Chen*

$These authors contributed equally to this work

*To whom correspondence should be addressed
ORCID Wei Chen: 0000-0002-6970-3455
Email: weichen@mtholyoke.edu

Address: Chemistry Department, Carr Laboratory, Mount Holyoke College, 50 College Street, South Hadley, Massachusetts 01075

Pages: 8
Figures: 6
Tables: 2
Videos: 2

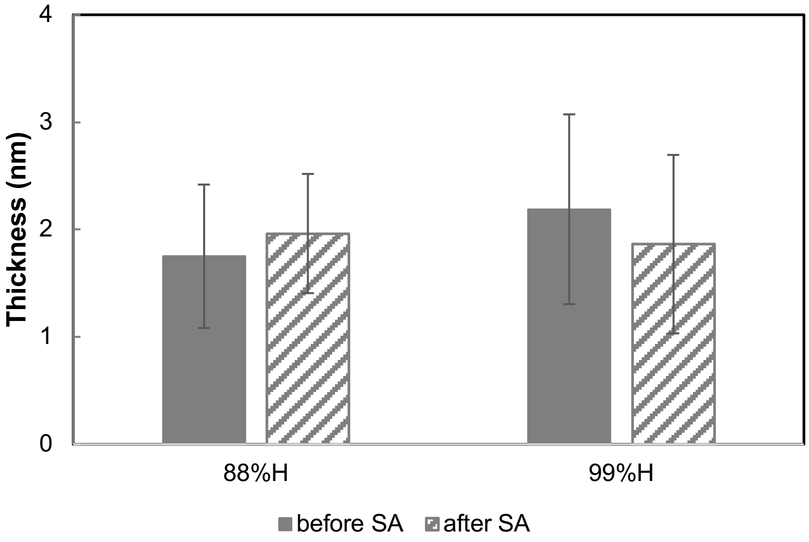

Figure 1S. Ellipsometric thickness of spin coated PVOH$^{88\%H}$ and PVOH$^{99\%H}$ fractal thin films on HMW PDMS before and after solvent annealing (SA) at room temperature for 1 h.

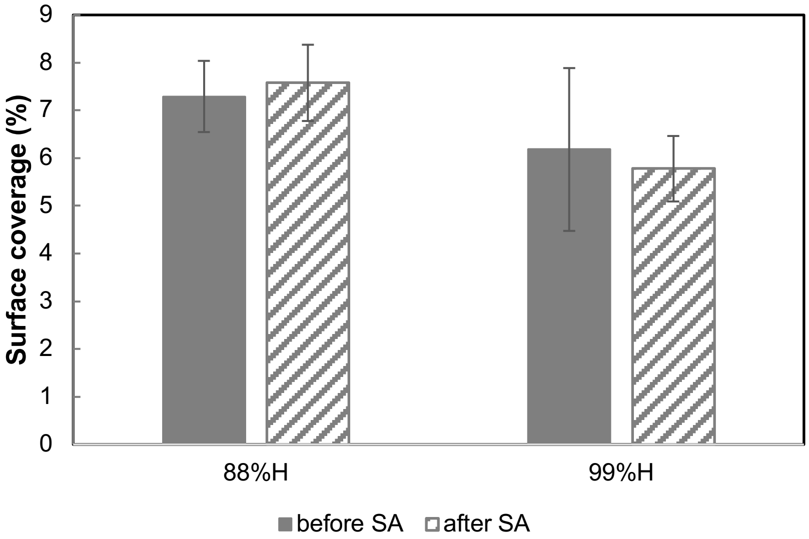

Figure 2S. Surface coverage of spin coated PVOH$^{88\%H}$ and PVOH$^{99\%H}$ fractal thin films on HMW PDMS before and after solvent annealing (SA) at room temperature for 1 h.

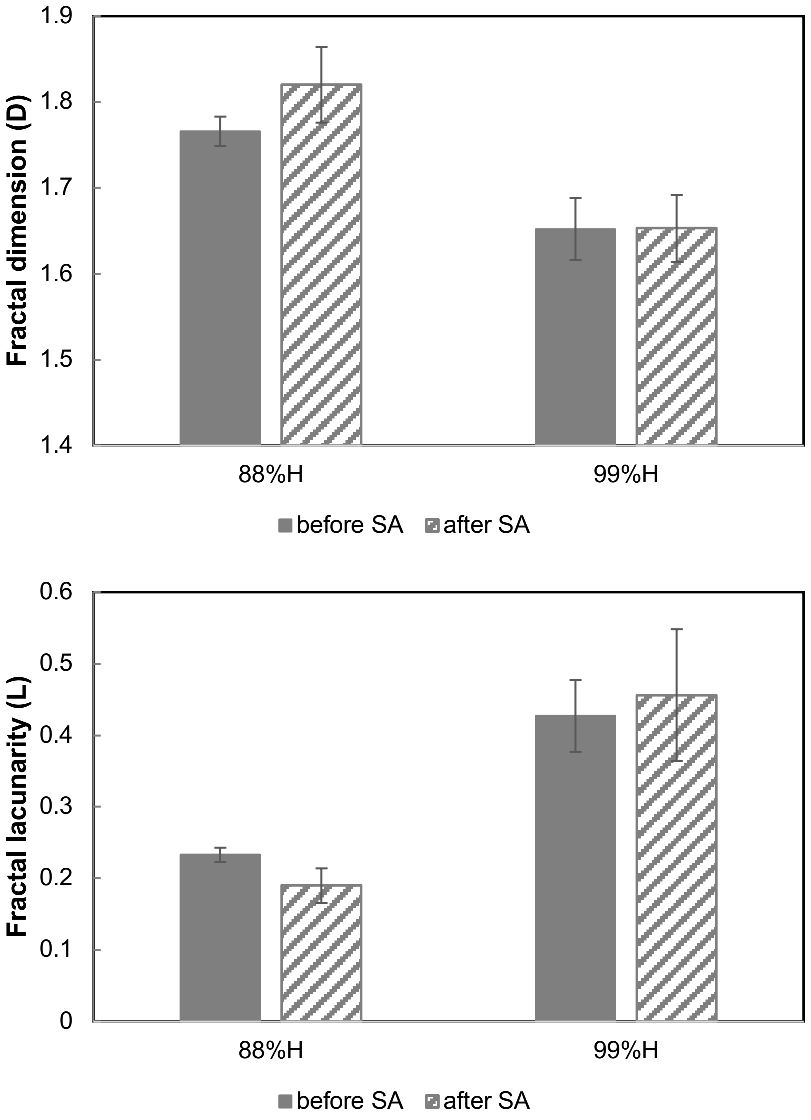

Figure 3S. Fractal dimension (top) and lacunarity (bottom) of spin coated $PVOH^{88\%H}$ and $PVOH^{99\%H}$ fractal thin films on HMW PDMS before and after solvent annealing (SA) at room temperature for 1 h.

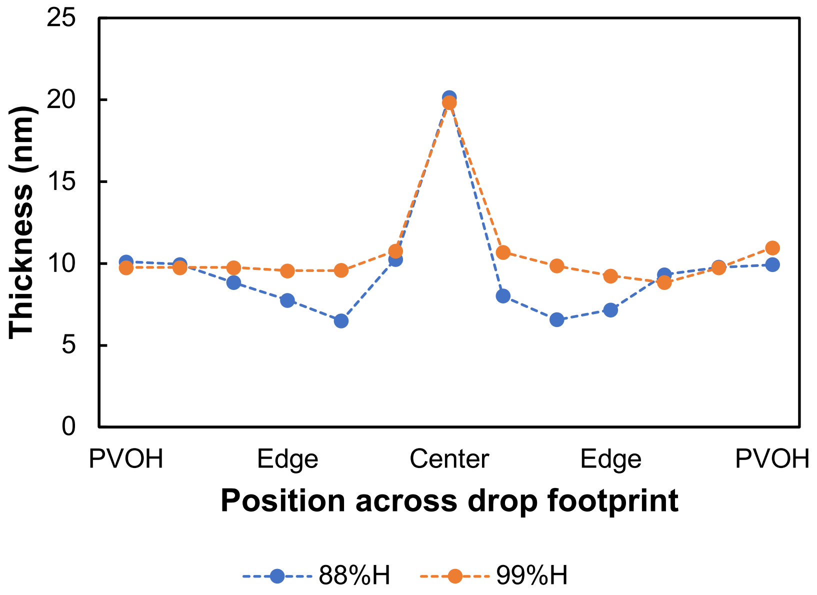

Figure 4S. Thickness profiles across footprint of 30 μL water drop, including the unperturbed PVOH background, on PVOH$^{88\%H}$ and PVOH$^{99\%H}$ films after water evaporation.

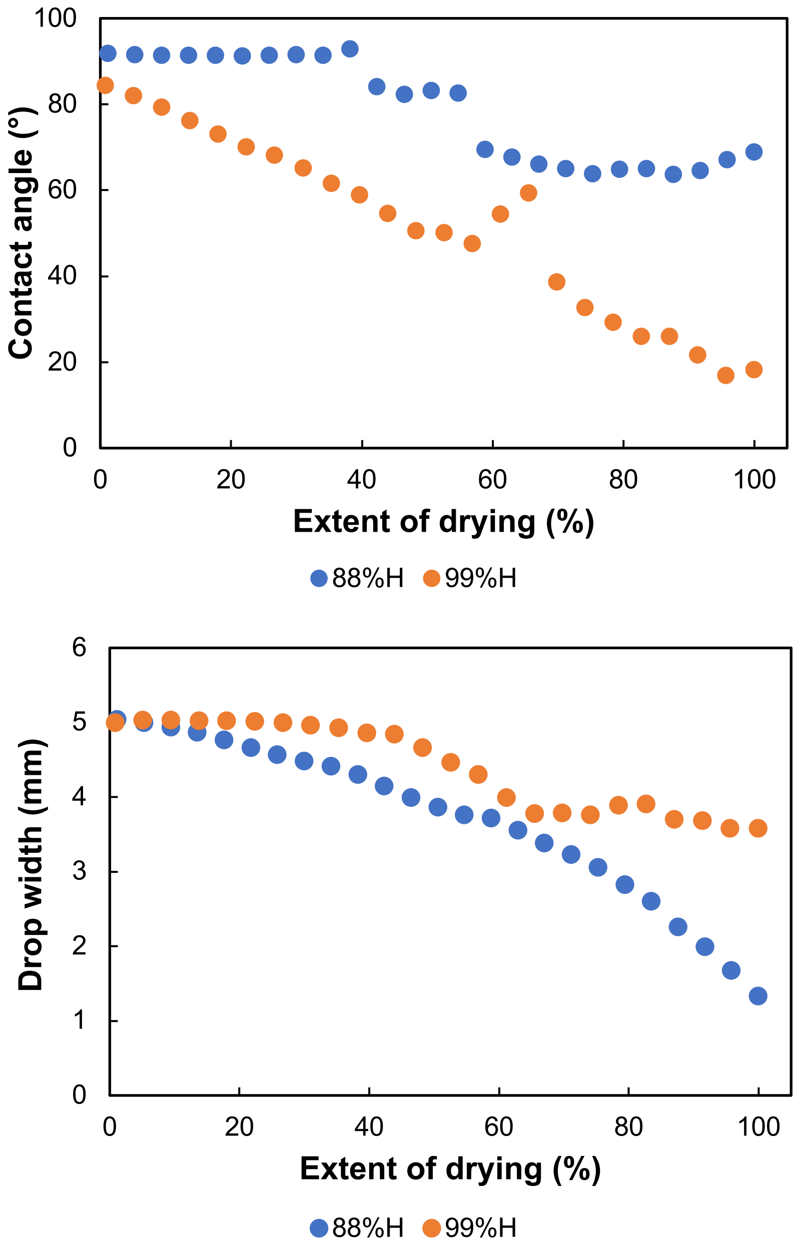

Figure 5S. Contact angle (top) and width (bottom) profiles of 30 μL waterdrop on PVOH$^{88\%H}$ and PVOH$^{99\%H}$ films as a function of extent of drying.

Table 1S. AFM images (size: 1.25 µm x 5 µm; data scale: 10 nm), advancing (θ_A) and receding (θ_R) contact angles, and surface coverage (%C) of statically adsorbed $PVOH^{99\%H}$ on HMW PDMS after in-situ crosslinking using glutaraldehyde for various amounts of time.

	**AFM**	**contact angle** θ_A/θ_R (°)	**%C**
control	N/A*	115±2/69±11	22±16%, ~6%**
1 min	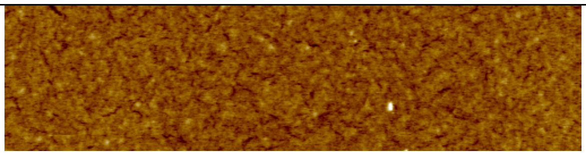	101±2/17±5	74±9%
5 min	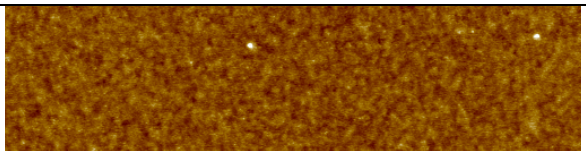	93±2/10±2	85±5%
10 min	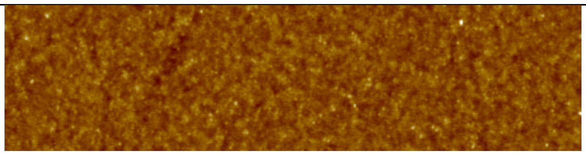	96±2/13±2	81±5%

*: see Figure 1
**: first value is based on average contact angle and second value is based on image analysis

Table 2S. AFM images (size: 1.25 µm x 5 µm; data scale: 10 nm), advancing (θ_A) and receding (θ_R) contact angles, and surface coverage (%C) of statically adsorbed $PVOH^{99\%H}$ on $PDMS^{2k}$ after in-situ crosslinking using glutaraldehyde for various amounts of time.

	**AFM**	**contact angle** θ_A/θ_R (°)	**%C**
control	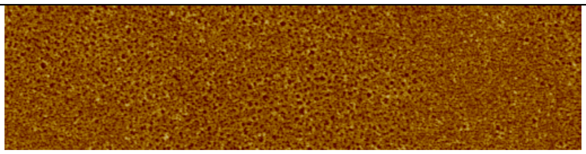	73±6/9±2	99±10%
1 min	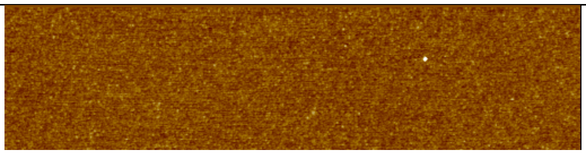	70±6/27±8	89±17%
5 min	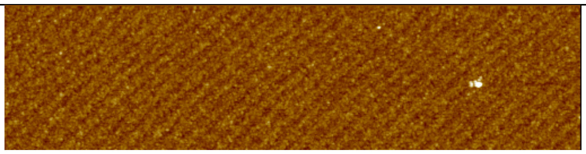	64±3/18±3	99±7%
10 min	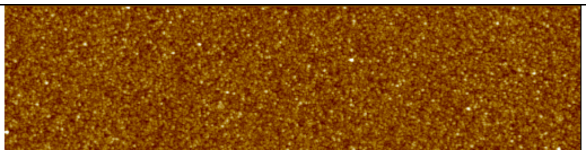	63±2/17±2	100±4%

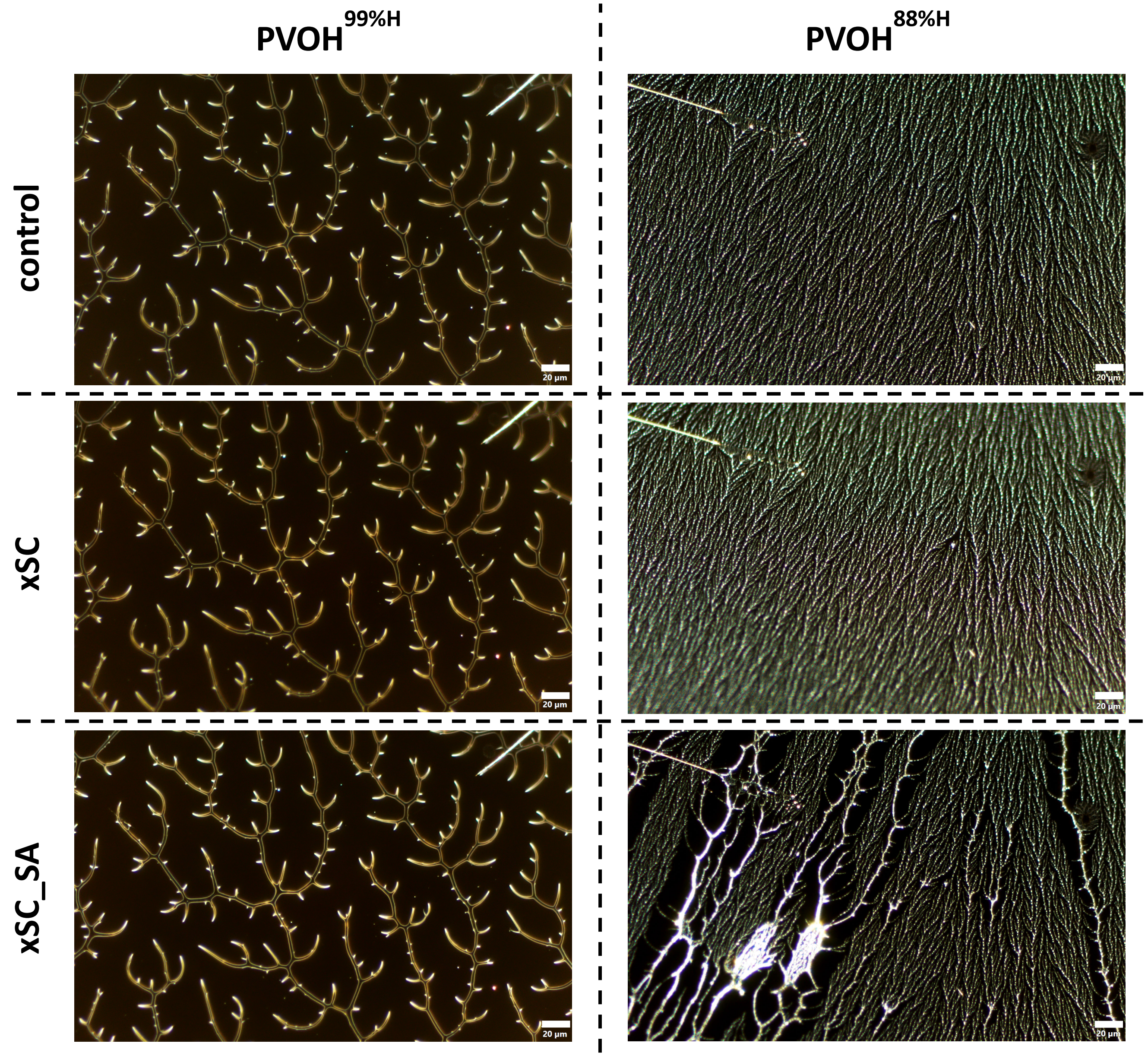

Figure 6S. Optical images of spin coated PVOH$^{99\%H}$ (left column) and PVOH$^{88\%H}$ (right column) on HMW PDMS (control, first row), after crosslinking with succinyl chloride at 70 °C for 1 h (xSC, second row), and after solvent annealing (SA) at room temperature for 1 h (xSC_SA, last row). Scale bars represent 20 µm.
